# Grasping the World: Object-Affordance Effect in Schizophrenia

**DOI:** 10.1155/2013/531938

**Published:** 2013-12-09

**Authors:** Jessica Sevos, Anne Grosselin, Jacques Pellet, Catherine Massoubre, Denis Brouillet

**Affiliations:** ^1^Department of Psychiatry, University Hospital of Saint-Etienne, Pavillon 52A, 25 Boulevard Pasteur, 42055 Saint-Etienne Cedex 2, France; ^2^Epsylon Laboratory, EA4556, Dynamics of Human Abilities and Health Behaviours, University of Montpellier III, Rue du Pr. Henri Serre, 34000 Montpellier, France

## Abstract

For schizophrenic patients, the world can appear as deprived of practical meaning, which normally emerges from sensory-motor experiences. However, no research has yet studied the integration between perception and action in this population. In this study, we hypothesize that patients, after having controlled the integrity of their visuospatial integration, would nevertheless present deficit in sensory-motor simulation. In this view, we compare patients to control subjects using two stimulus-response compatibility (SRC) tasks. Experiment 1 is performed to ensure that visuo-spatial integration is not impaired (Simon Effect). Experiment 2 replicates a study from Tucker and Ellis (1998) to explore the existence of sensory-motor compatibility between stimulus and response (Object Affordance). In control subjects, the SRC effect appears in both experiments. In schizophrenic patients, it appears only when stimuli and responses share the same spatial localization. This loss of automatic sensory-motor simulation could emerge from a lack of relation between the object and the subject's environment.

## 1. Introduction

For the embodied theories of cognition, it is largely accepted that cognitive processes in healthy subjects are deeply rooted in the sensory-motor systems. The cognitive functioning is carried out dynamically, throughout a context, a situation, a task, and a body status [1]. More particularly, perception and action are inseparable; indeed, perception has to be understood as an action guided towards its aim.

This embodied view of mind is in accordance with Merleau-Ponty's [2] phenomenology of perception in which the organism and environment are coupled. For him, the body's actions are the conditions for entering into a cognitive relationship with the world. In relation to both motor and perceptual abilities of the subject, the objects of the world are perceived as “practicable” objects.

In the embodied theories of cognition, the concept of affordance, originally introduced by Gibson [3] in 1977, has a key role. Indeed, affordances depend on the context, perceptual characteristics of objects and on the body; objects, and environment are seen in terms of opportunity of actions [4]. The visual representation of an object comes from the binding between its visual properties and related action potentialities via sensory-motor simulations. Both behavioral [5–9] and brain imaging studies [10–12] indicate that the representation of perceived objects incorporates motor information. This action-perception binding thus allows all living being to automatically perceive an object as a support for its action.

To explore this idea, Tucker and Ellis [13] used a stimulus response compatibility (SRC) paradigm. Tasks using this paradigm highlight that performances not only depend on the stimuli or response modalities but also on the way these elements combine themselves together. According to this paradigm, when the stimulus and the response share the same properties, response times are shorter than when they do not: this is the “SRC effect” [14]. In their study, Tucker and Ellis [13] focused on the action potentiation effect when perceiving graspable objects. They asked participants to answer, with their right or left hand, to the vertical orientation (upright or inverted) of a graspable object presented in the middle of the screen. They found that responses were faster when there is a compatibility between the graspable part of the object and the response hand (right-hand response with the handle oriented to the right hand side or left-hand response with the handle oriented to the left side) than when there is incompatibility. In this experimental context, Tucker and Ellis [13] postulated that the intrinsic properties of the objects emerge from perception without action instructions: the “object-based affordance effect” [10]. In other words, seen objects automatically potentiate components of the actions they afford.

Another well-known effect can be found using an SRC paradigm, based here on the congruency between the spatial localization of the stimulus and the motor response: the “Simon effect” [15]. Due to a visuo-spatial integration, response times are faster when the stimulus is presented on the right hand side of the screen and the response is given by the right hand (the same congruency is found for the left hand side/left hand).

Certain authors postulate that the object-based affordance effect, shown by Tucker and Ellis [13], could be due to a spatial Simon effect. For them, the visual asymmetry of stimuli would tend to capture visual attention and create a visuo-spatial compatibility between the spatial localization of the stimulus and motor response side [16, 17]. Conversely, for Symes et al. [18] or Buccino et al. [19], object-based affordance could clearly be distinguished from the spatial Simon effect. For them, automatic responses would be guided by the intrinsic properties of the objects and not by their spatial localization [10].

In psychopathology of schizophrenia, symptoms such as derealization (external world feels strange or transformed) and depersonalization (feeling of being strange or having one's own body transformed) are encountered [20, 21], even if new treatments improve frequently these symptoms. More recently, authors that build on the work of phenomenological philosophers such as Heiddeger [22], describe schizophrenia's main disorder as a fundamental disorder of self-embodiment, relating to a loss of a sense of familiarity with the external world [23–25]. Even if patients presenting with schizophrenia are able to identify objects in their environment, they are frequently not able to use them without adaptive strategies. These considerations offer a new reading of impaired subjective experiences or of disturbed social interactions [26, 27]. Usually, the body allows the subject to be adapted to the world and to others, throughout an embodied common sense and certain “know-how” [28]. However, patients may sometimes behave as if they have a body without a spirit or a spirit without a body; this may question the embodiment of the self, of the object relation, or social relationships in this population [24].

According to Sass and Parnas [29], things usually tacit for healthy subjects would become explicit for patients. Sensory-motor processes, normally automatic, would become available for conscious introspection in this population, due to a fragmentation of automatic perceptual and motor schemes [25]. This could lead to a loss of the feeling of inhabiting their own perception, actions, bodily sensations, and also to a disturbance of basic self-awareness in schizophrenia [25, 30]. Thus, it would become difficult for patients to “organize the experiential world in accordance with needs and wishes, thereby giving objects their affordance” [30]. For patients, the world can appear as deprived of practical meaning, which normally emerges from sensory-motor experiences. However, can a phenomenon such as affordance still emerge if subject and environment are not interlinked anymore?

In this population of patients, no research has yet specifically studied the automatic integration between perception and action, which constitute the foundation of an embodied cognition. However, on an experimental level, a few cognitive studies take an interest in the general question of perception and action in schizophrenia. Enticott et al. [31] showed a decrease of motor-evoked potentials in schizophrenic patients, when seeing actions compared to healthy subjects. Franck et al. [32] demonstrated that it is more difficult for patients than for control subjects to recognize their movements as their own. Delerue and Boucart [33] are the only authors suggesting impairment for the detection of object-affordances in schizophrenic patients. Using an eye-tracking task, they asked subjects to either name the objects or the actions inferred by the objects presented. Results showed that healthy subjects explored the whole object in the action-naming task and only the part needed for identification in the object-naming task, whereas patients used the same visual exploration patterns for both tasks.

Nevertheless, the visuo-spatial compatibility is not taken into account in these studies. To our knowledge, the only study having used the Simon paradigm in schizophrenia showed a visuo-spatial compatibility specifically when the stimuli were presented in the left hemifield [34].

In this paper, we propose to use the SRC paradigm, classically used in an embodied perspective of cognition, in order to explore the integration between perception and action in this pathology. Such a study implies to control the integrity of visuo-spatial integration and, in order to achieve this, we use a Simon task (Experiment 1). In Experiment 2, we explore the hypothesis that schizophrenic patients could have a deficit in sensory-motor simulation during perception of graspable objects, in contrast to healthy subjects. In schizophrenia, the sensory-motor simulation impairments could emerge from a lack of connection between the object and the subject's environment without impairment in visuo-spatial integration.

## 2. Experiment 1

### 2.1. Participants

Seventeen schizophrenic patients, recruited from the University Hospital of Saint-Etienne, participated in the study. All were outpatients living in their own accommodation with various psychosocial or professional activities. The inclusion criteria were a DSM-IV diagnosis of schizophrenia [35]; there was no change in antipsychotic medication and clinical status within four weeks prior to the study. All patients were assessed by the same psychiatrist with the positive and negative syndrome scale (PANSS) [36], which showed the predominance of a positive syndrome on 9 patients and of a negative syndrome on 8 patients.

Seventeen healthy control subjects recruited via advertisement in the local newspaper were matched to the schizophrenic subjects on age, years of education, and score on a modified Edinburgh Handedness Inventory [37] (see Table 1 for mean group scores and statistical comparisons).

The noninclusion criteria for all participants were (1) a diagnosis of neurological brain disorder or head trauma with loss of consciousness, (2) a mental retardation, (3) history of substance abuse over the last 6 months, and (4) an Edinburgh score inferior to 14.

The study was approved by the local Ethics Committee from Saint-Etienne, and an informed written consent was obtained from all participants.

### 2.2. Apparatus and Materials

In this task, we used the computer-based attention battery TAP [38]. In this task, arrows, pointing left or right, appear either on the left or right-hand side of a fixation point.

### 2.3. Design and Procedure

Each participant carried out a block of 60 trials. Subjects had to press the response key as fast and accurately as possible with their right or left index, according to the direction of the arrow, without taking into consideration its side of appearance. White arrows were presented on a black screen, either pointed to the same side of its appearance (compatibility) or to the opposite side (incompatibility). The response keys were situated 15 cm apart and 20 cm in front of the screen. The order of trials was randomized between subjects.

Before the beginning of the experiment, subjects were seated with their head 50 cm in front of the screen. Each experimental trial started with a fixation point of 1500 ms, immediately followed by an arrow presented on a black screen. The stimulus was flashed on the screen every 3000 ms. If no answer was given before 2000 ms ended, the message “no response” appeared for an extra 2000 ms. Each participant received 7 practice trials.

### 2.4. Results

For all conditions, participants responded during the first 2000 ms. No differences on mean error rates were found between patients and control subjects (see Table 2).

The mean response times (RT) and SD were calculated for each subject; RT above 2 SD of their own individual mean were eliminated (under 5%).

**Figure 1:**
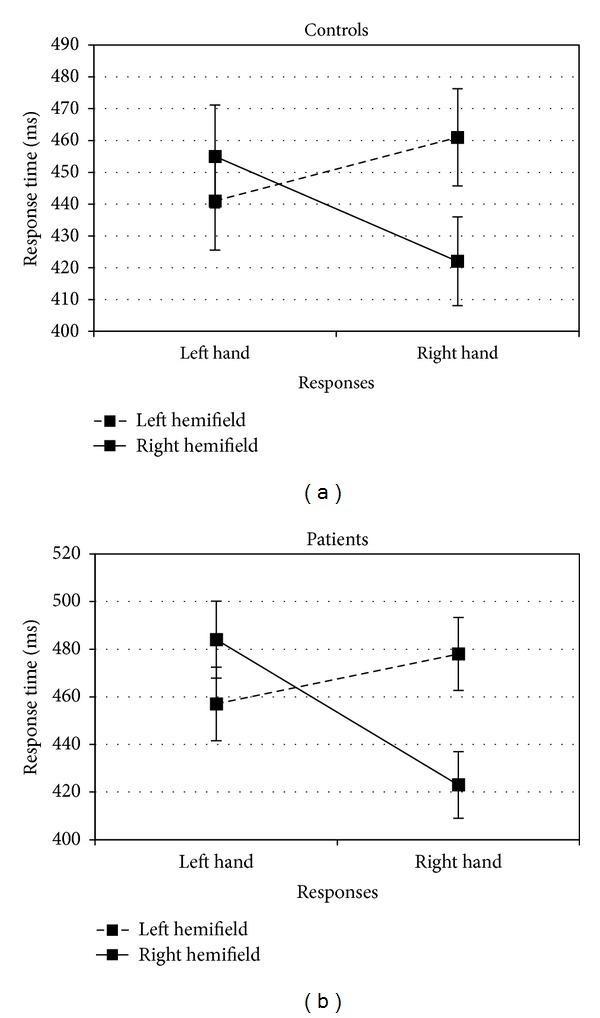
Mean response times for Experiment 1 as a function of groups (controls or patients), hemifield (left or right), and responses (left or right hand).

An analysis of variance (ANOVA) was conducted on the participants' data with *Group *(controls or patients) as between subject factor, and *Response* (left or right hand) and *Field* (left or right hemifield) as within subject factor. Patients (461 ms) were not significantly slower than controls (445 ms) (*F*_(1,32)_ = 0.570, *η* ^2^ = 0.01, *P* = .461). There was a main effect of *Response *(*F*_(1,32)_ = 9.169, *η* ^2^ = 0.22, *P* = .008), but the *Response* × *Group* interaction was not significant (*F*_(1,32)_ = 1.966, *η* ^2^ = 0.05, *P* = .180). The main effect of *Field* was significant (*F*_(1,32)_ = 4.753, *η* ^2^ = 0.12, *P* = .045) with faster response times when the arrows were presented in the right hemifield (446 ms) compared to the left (459 ms). The* Field* × *Group* interaction was not significant (*F*_(1,32)_ < 1). The 2-way interaction between *Field *and* Response* (which measures the Simon effect) was found (*F*_(1,32)_ = 31.994, *η* ^2^ = 0.49, *P* = .001). Right-hand responses were faster when the object appeared in the right hemifield (423 ms) rather than in the left (470 ms; *t*_(16)_ = −5.324, *η* ^2^ = 0.63, *P* = .001). Similarly, left-hand responses were faster when the object appeared in the left hemifield (449 ms) rather than in the right (470 ms; *t*_(16)_ = 2.546, *η* ^2^ = 0.28, *P* = .016). In addition, this effect was not significantly modified by group (*F*_(1,32)_ = 1.013, *η* ^2^ = 0.03, *P* = .329) (see Figure 1).

In this experiment, control subjects and patients showed the same pattern of responses; their RTs are faster when there was compatibility between the spatial localization of the stimulus and the motor response. After having controlled the integrity of visuo-spatial integration in schizophrenic group, we explore, in Experiment 2, if they present impairment in sensory-motor simulation.

## 3. Experiment 2

### 3.1. Participants

All of the patients and control subjects from the first experiment also carried out this second one; 3 new patients and 3 new control subjects were contacted in order to expand the sample size, which helps to boost statistical reliability. The 2 groups were still matched in ages, years of education scores, and handedness (see Table 3 for mean group scores and statistical comparisons).

New patients were assessed by the same psychiatrist with the positive and negative syndrome scale (PANSS) [36]. The group as a whole showed the predominance of a positive syndrome on 11 patients and of a negative syndrome on 9 patients.

### 3.2. Apparatus and Materials

**Figure 2:**
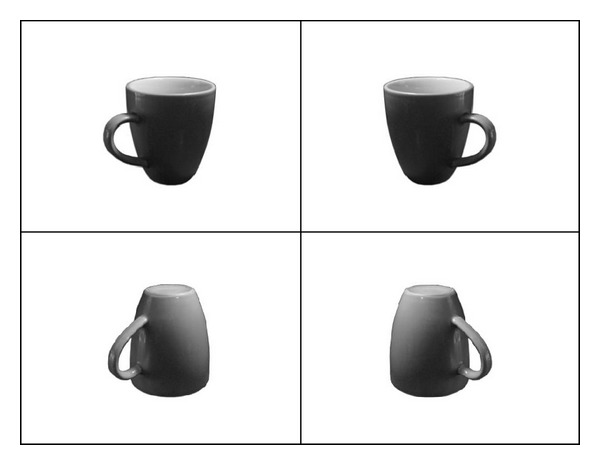
Examples of the stimuli used in Experiment 2: left orientation, upright; right orientation, upright; left orientation, inverted; right orientation, inverted.

Black and white photos of 22 objects were used (see Appendix). All of the objects were graspable by one hand and were photographed in 2 horizontal orientations (compatible either with a right or left hand grasp) and 2 vertical orientations (upright or inverted) (see Figure 2). Consequently, 88 pictures were presented in the middle of a computer screen. On the screen, all average size was 512 × 384 pixels, maintaining the proportions of each object at a distance of 50 cm.

### 3.3. Design and Procedure

Each participant carried out 2 blocks of 88 trials with a 3-minute pause between each block. These blocks differed in terms of response mapping (right hand-upright/left hand-inverted and left hand-upright/right hand-inverted) and were counterbalanced between subjects. They had to press the response key as fast and accurately as possible, with their right or left index, according to the vertical orientation of the objects (upright or inverted). Subjects kept their right finger on a right response key and their left finger on a left response key during the whole experiment. Depending on the situation, the response hand could be on the same side as the graspable part of the object (compatibility) or on the opposite side (incompatibility). On a standard AZERTY keyboard, the response keys were situated 15 cm apart and 20 cm in front of the screen. The order of trials was randomized between subjects.

Before the beginning of the experiment, subjects were seated with their head 50 cm in front of the screen. Every photo was shown to the participants, in order to be sure that they recognized the object, in upright or inverted orientation. Each experimental trial started with a 1500 ms fixation point, immediately followed by an object presented on a white screen. The stimulus stayed on the screen until an answer was given or during 3000 ms if not. Participants were given feedback on errors (short tone). Each participant received 16 practice trials (with other photos of objects) before each block.

### 3.4. Results

For all conditions, participants responded during this given time of 3000 ms. No differences on mean error rates were found between patients and control subjects (see Table 4).

The mean response times (RT) and SD were calculated for each subject; RT above 2 SD of their own individual mean were eliminated (under 5%).

An analysis of variance (ANOVA) was conducted on the participants' data with *Group* (controls or patients) as between subject factor, and *Response* (left or right hand) and object *Orientation* (left or right) as within subject factors.

**Figure 3:**
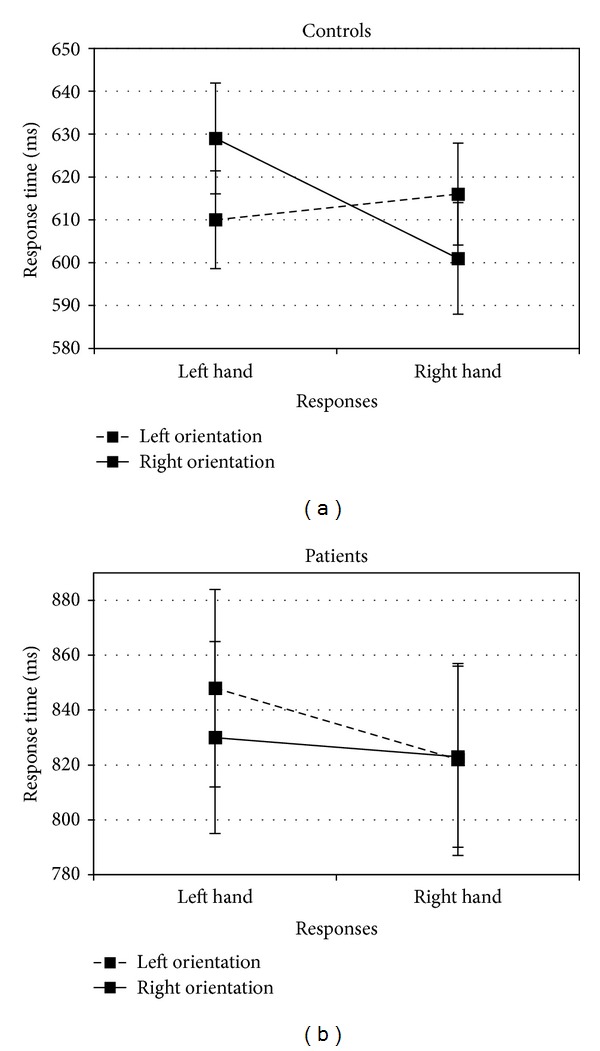
Mean response times for Experiment 2 as a function of groups (controls or patients), orientations (left or right), and responses (left or right hand).

Globally, patients showed increased RT compared with controls as reflected by a significant main effect of group (*F*_(1,38)_ = 31.77, *η* ^2^ = 0.45, *P* = .001). We also found a main effect of *Response* (*F*_(1,38)_ = 7.347, *η* ^2^ = 0.16, *P* = .014) with longer RT when subjects gave left hand responses (729 ms) in comparison to right hand responses (716 ms). However, the *Response* × *Group* interaction was not significant (*F*_(1,38)_ < 1), reflecting similar costs produced by left hand responses across groups. The main effect of object *Orientation* was not significant (*F*_(1,38)_ = .733, *η* ^2^ = 0.01, *P* = .403) in the same way as the *Orientation* × *Group* interaction (*F*_(1,38)_ = .898, *η* ^2^ = 0.02, *P* = .355). Whilst the *Response* × *Orientation* interaction (which measures the compatibility effect) was not significant (*F*_(1,38)_ = 1.520, *η* ^2^ = 0.03, *P* = .233), the most interesting result was the three-way *Group* × *Response* × *Orientation* interaction (*F*_(1,38)_ = 11.956, *η* ^2^ = 0.23, *P* = .003) (see Figure 3). In the control group, right-hand responses were faster when the orientation of the object was also to the right rather than to the left (*t*_(19)_ = −2.567, *η* ^2^ = 0.25, *P* = .019). Similarly, left-hand responses were faster when the orientation of the object was also to the left rather than to the right (*t*_(19)_ = 3.151, *η* ^2^ = 0.34, *P* = .005). The *Response* × *Orientation* two-way interaction was significant (*F*_(1,19)_ = 25.962, *η* ^2^ = 0.57, *P* = .001). In the patient group, there was no such significant difference for right-handed responses to objects oriented to the right or to the left (*t*_(19)_ < 1). Left handed responses to objects oriented to the right were actually faster than the responses to objects oriented to the left (*t*_(19)_ = −1.845, *η* ^2^ = 0.15, *P* = .081). The *Response* × *Orientation* two-way interaction was not significant (*F*_(1,19)_ = 2.114, *η* ^2^ = 0.09, *P* = .162).

To control a possible tiredness effect due to task in the patient group, we compared RT and errors between the first 44 trials of the first block and the last 44 trials of the second block. There was no difference in RT between the first 44 trials (843 ms) and the last 44 trials (842 ms) (*t*_(19)_ < 1). This pattern was also found for the error rates (resp. 4,8% and 5%; *t*_(19)_ < 1).

In this second experiment, we found the same results as Tucker and Ellis [13] on control subjects' RT. Their responses were faster when there was compatibility between the orientation of the prehensile part of common graspable objects and the response hand. However, this object-based affordance effect did not emerge in the schizophrenic group.

## 4. General Discussion

The aim of these tasks was to investigate if schizophrenic patients have a deficit in sensory-motor simulation without impairment in visuo-spatial integration. In Experiment 1, we demonstrated that the compatibility between the spatial localization of a stimulus and of the motor response leaded to faster response times, even for schizophrenic patients. Experiment 2 replicated the results of Tucker and Ellis [13] on control subjects. Responses were faster when there was compatibility between the orientation of common graspable objects and the response hand. However, this object-based affordance effect was not found in the schizophrenic group.

These results seem particularly interesting; for schizophrenic patients, the stimulus response compatibility effect does not appear for every condition (Simon-task and affordance-task). Indeed, our results show that, in this population, the SRC effect appears only when stimulus and response share a visuo-spatial localization. This Simon effect is already partly found in the study of Gastaldo et al. [34] which focuses only on patients with negative symptoms. They show a spatial compatibility only when the stimuli are presented in the left hemifield. Let us underline that this study focuses on abnormalities of visual attention, frequently reported in patients [39], rather than on the Simon effect. Our results demonstrate that, for both positive and negative symptom patients, the Simon effect appears in both hemifields. Moreover, there is no group difference in RT measures, as already found by Behrwind et al. [40] in a visuo-spatial version of SRC tasks. In the schizophrenic group, the visuo-spatial integration seems to be automatic, in the same way as in control subjects.

However, even for patients with a low level of symptom severity, our results do not bring to light the object-affordance effect. During the task replicating Tucker and Ellis' experiment [13], our results show an increase of patients' RT in comparison to those of the control group. This increase is not due to a possible tiredness effect. However, it could reflect an absence of automatic simulation which normally appears when stimulus and response share sensory-motor characteristics. In the patient group, it would seem that the automatic binding between perception and action does not take place. Thus, the increased RT could reflect the implementation of controlled processes more costly in attentional resources.

Our 2 experiments, using the SRC paradigm, seem to distinguish the spatial Simon effect from an object-based affordance effect, as already found by Symes et al. [18] and Buccino et al. [19]. However, for other authors [16, 17], the perceptive asymmetry of manipulable objects would virtually make the graspable part seem closer to the hand; this perceptive salience would attract visual attention. Buccino et al. [19] use a cup with a broken handle, which has the same visual asymmetry as an undamaged cup. They show that this broken cup does not trigger off the premotor activity linked to the perception of an undamaged cup. In this case, a broken cup would not represent a typical graspable object, underlying the importance of the pragmatic role of an object. If object-affordance effect only emerges from a visual asymmetry, patients in our study should have similar results to control subjects on the Tucker and Ellis task [13]. The contrast between the patterns of results of the two experiments in schizophrenic patients, leads us to think that the implementation of sensory-motor simulation is a different effect from visual saliency.

Consequently, if patients present sensory-motor integration impairment, the relationship between the object's features and the action to carry out cannot automatically be “built”. Alternative explanations of lack of interest or deficit in attention could not account for the reduced affordance-effect in patients with schizophrenia because they do not make more errors than control subjects. However, making the action more specific to the patient's needs and wishes or reinforcing the purpose of the action could help create this automatic link between perception and action.

To conclude, the results of our study on object-affordance seem to us, even if more confirmative studies are needed, a promising way to enlarge knowledge on cognitive disturbances of embodiment in schizophrenia. They could also highlight a cognitive basis of phenomenon like depersonalization and derealization, that is, perturbations of links between patients and their environment. These considerations could join the following Stanghellini's quote [41]: “if my body-based involvement in the world is switched off, my grasp onto the world will fade away too”.

## Figures and Tables

**Table 1:** Age, years of education, and Edinburgh scores (SD) comparison between patients and control subjects for Experiment 1.

	Controls (*N* = 17)	Patients (*N* = 17)	*t*-test; *P* value
Age (years)	33.9 (8.2)	36.2 (6.4)	*t* = .902, *P* = .204
Education (years)	12.8 (2.3)	12.2 (2)	*t* = − .723, *P* = .963
Edinburgh score (/20)	18.9 (145)	18.1 (1.7)	*t* = 1.292, *P* = .215

**Table 2:** Error rates (SD) comparison between groups in Experiment 1.

		Patients	Controls	*t*-test; *P* value
Compatibility	Right hand	0.2 (0.5)	0.6 (0.7)	*t* = − 1.692, *P* = .110
	Left hand	0.2 (0.6)	0.6 (1)	*t* = − 1.191, *P* = .251
Incompatibility	Right hand	0.6 (0.9)	0.2 (0.8)	*t* = 1.383, *P* = .186
	Left hand	0.2 (0.6)	0.4 (0.6)	*t* = − .523, *P* = .608

**Table 3:** Age, years of education, and Edinburgh scores (SD) comparison between patients and control subjects for Experiment 2.

	Controls (*N* = 20)	Patients (*N* = 20)	*t*-test; *P* value
Age (years)	34.1 (8.4)	36.2 (5.9)	*t* = .935, *P* = .356
Education (years)	12.8 (2.1)	11.9 (2.8)	*t* = − 1.228, *P* = .227
Edinburgh score (/20)	18.3 (1.7)	18.7 (1.5)	*t* = .779, *P* = .441

**Table 4:** Error rates (SD) comparison between groups in Experiment 2.

		Patients	Controls	*t*-test; *P* value
Compatibility	Right hand	4.4 (3.1)	3.8 (2.3)	*t* = .765, *P* = .449
	Left hand	4.2 (3.5)	2.6 (2.5)	*t* = 1.704, *P* = .096
Incompatibility	Right hand	4.5 (3.7)	4.3 (3.2)	*t* = .183, *P* = .856
	Left hand	4.1 (2.9)	4.3 (2.5)	*t* = − .288, *P* = .775

## Appendix

### Objects Used in Experiment 1

Watering plant
Handled dustpan
Bottle of detergent
Bottle of detergent
Kettle
Coffee pot
Coffee pot
Saucepan
Saucepan
Knife
Knife
Iron
Whisk
Mug
Mug
Sieve
Sieve
Watering can
Frying pan
Frying pan
Small jug
Remote control.
